# A novel role of C-terminus in introducing a functionally flexible structure critical for the biological activity of botulinum neurotoxin

**DOI:** 10.1038/s41598-018-26764-z

**Published:** 2018-06-11

**Authors:** Thomas M. Feltrup, Kruti Patel, Raj Kumar, Shuowei Cai, Bal Ram Singh

**Affiliations:** 1 Department of Chemistry & Biochemistry, University of Massachusetts Dartmouth, North Dartmouth, MA 02747 USA; 2 Botulinum Research Center, Institute of Advanced Sciences, North Dartmouth, MA 02747 USA

## Abstract

Botulinum neurotoxin (BoNT) is responsible for botulism, a clinical condition resulting in flaccid muscle paralysis and potentially death. The light chain is responsible for its intracellular toxicity through its endopeptidase activity. Available crystal structures of BoNT/A light chains (LCA) are based on various truncated versions (tLCA) of the full-length LCA (fLCA) and do not necessarily reflect the true structure of LCA in solution. The understanding of the mechanism of action, longevity of intoxication, and an improved development of endopeptidase inhibitors are dependent on first having a better insight into the structure of LCA in solution. Using an array of biophysical techniques, we report that the fLCA structure is significantly more flexible than tLCA in solution, which may be responsible for its dramatically higher enzymatic activity. This seems to be achieved by a much stronger, more rapid binding to substrate (SNAP-25) of the fLCA compared to tLCA. These results suggest that the C-terminus of LCA plays a critical role in introducing a flexible structure, which is essential for its biological function. This is the first report of such a massive structural role of the C-terminus of a protein being critical for maintaining a functional state.

## Introduction

Botulinum neurotoxin (BoNT) is a potent molecule (mouse LD50 of 0.1–1 ng/kg)^1^ with seven distinct serotypes (labeled A-G) responsible for botulism, a clinical condition which results in flaccid muscle paralysis and can potentially lead to death. The BoNT molecule is composed of two subunits – the heavy chain (HC) and light chain (LC). The C-terminus of the HC targets and binds the polysialoganglioside receptors on presynaptic nerve terminal and the N-terminus assists in the translocation of the LC^1,2^. Once inside the cell, the LC acts as an endopeptidase and targets a specific part of the SNARE complex (soluble N-ethylmaleimide sensitive factor attachment protein receptors), specifically SNAP-25 for BoNT/A^3^. This LC is the subject of interest since it is highly specific, long-lasting in cells, and high-resolution structural data in solution has not been achieved.

Botulinum neurotoxin possesses evolutionary traits for targeting the exocytosis process that leads to the blockade of acetylcholine release leading to the muscle paralysis^4^. The key component of BoNT for intracellular disruption of the exocytosis process is its LC, which lasts inside the neuronal cells for several months to maintain continued muscle paralysis, a feature virtually unheard of in the biological world.

Identifying the key components of the structure of BoNT/A LC (LCA) in solution is a critical factor in better understanding the mechanism of action, longevity of intoxication, and assisting in the development of potent inhibitors of the endopeptidase activity. Mostly for the reason of solubility, the recombinant LCA currently being used for crystallization (based on the crystal structures of LCA available in the Protein Data Bank, PDB) are based on a variety of truncated versions of the native LCA (typically truncated at the C-terminus to yield LCA 1–424) and may not necessarily reflect the true structure of the full-length LCA (448 residues)^5,6^, which are being used for structural studies^7^ or inhibitor development^8–16^. Solubility issues and flexibility due to the presence of the C-terminus in fLCA have proved difficult to overcome in crystallization and have not allowed researchers to solve a crystal structure. While crystal structures of the full-length LCA are not available due to the solubility problems, the latter is not necessarily the only issue in the case of LCA. Previous studies have shown fLCA exists in a catalytically optimum PRIME (PRe-Imminent Molten Globule Enzyme) conformation consistent with an expanded and loosened structure in solution at 37 °C which would not be crystallizable under normal conditions^5,6^.

Solubility of variants of LCA was shown to be dependent on the C-terminus. Deletion and mutation mapping of the C-terminus demonstrated the most soluble variants of LCA were LCA-425 and LCA-418 while fLCA was subject to poor stability^7,17,18^. Studies have demonstrated that the LCA-425 model^18^ and LCA-9–415 model^16^ were soluble at 4 °C for several weeks with only minor degradation in the absence of salts and glycerol. One report demonstrated the LCA-425 model was purified at the highest yields and the LCA-418 model was also very stable and able to be concentrated up to 40 mg/mL^18^. The increased solubility and stability of these variants, especially LCA-425, are particularly attractive to researchers developing inhibitors of the BoNT/A endopeptidase activity.

The structure of LCA in aqueous solution is also important to selectively bind to its substrate, SNAP-25, where cleavage of the SNAP-25 results in a blockade of neurotransmitter release. For the mechanism of this selectivity, as well as identification of inhibitors to this selective binding, accurate protein folding of the LCA is important not only in a free enzyme state, but also in a substrate-bound state.

In this study, the solution structure of fLCA (448 residues) and a tLCA (424 residues) was probed using a series of spectroscopic techniques with LCAs in free and substrate-bound forms. The endopeptidase activity with a peptide and full-length substrate was correlated with the semi-resolved structural information to better explain the differences between fLCA and tLCA. Identifying these differences is an important step to unravel the structural and functional differences between tLCA and fLCA. In addition, the understanding gained from this work can be applied not only in designing better countermeasures against botulism, but also in understanding the mode of biological functions of the LCA, and longevity of LCA inside the cells. Substantial structural and functional differences were observed between fLCA and tLCA, suggesting a major role of the structural flexibility and dynamics in its biochemical function. Although published crystal structures displayed the uniqueness of LCA and explained the interaction with its substrate, it is unable to explain various questions related to its structure, stability, solubility, and function. Differences between crystal and solution structure in earlier work^5,6,19,20^ and this work indicate an urgent need to get a fully resolved solution structure and conformational dynamics of the full-length LCA.

## Results

### LCA Endopeptidase activity with Peptide and Full-length Substrates

**Figure 1:**
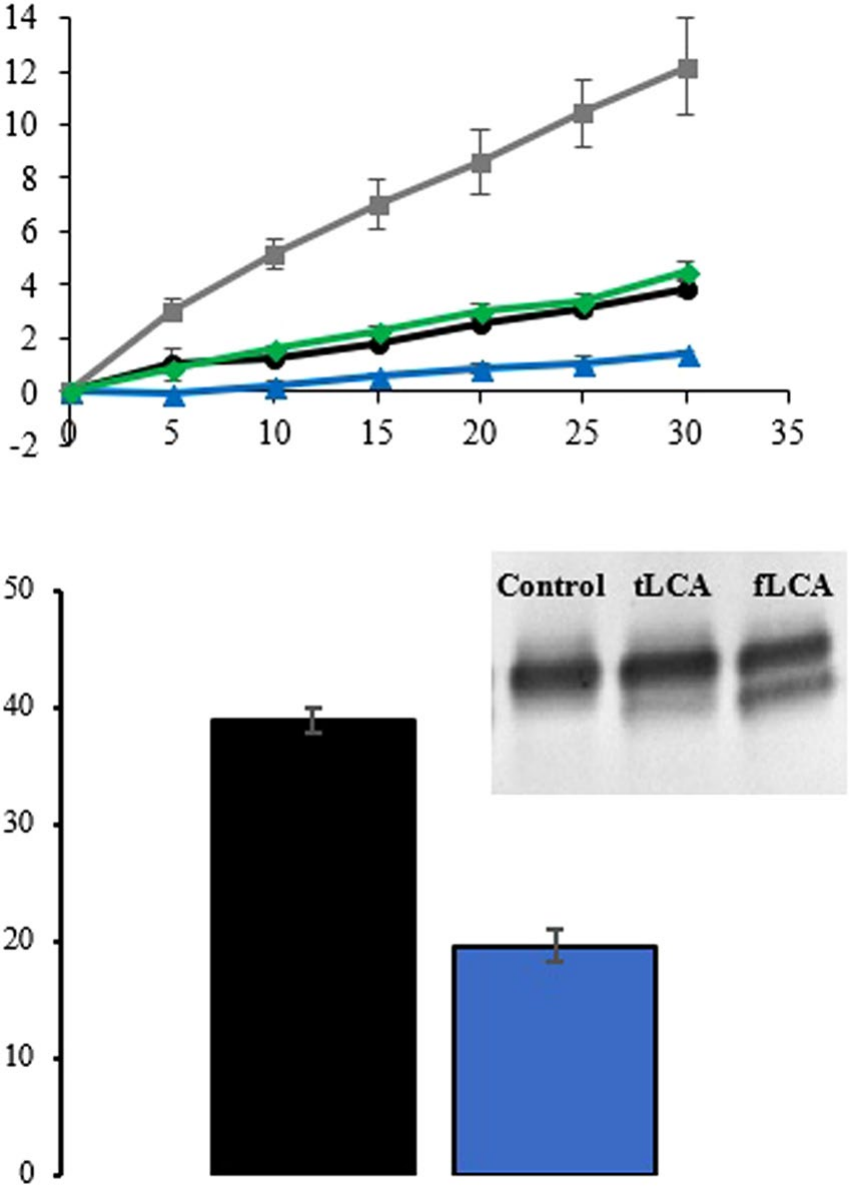
(**a**) Cleavage of peptide substrate by fLCA and tLCA. 4 μM Nutide was used as a substrate with 50 nM LCA over 30 min at 25 °C and 37 °C. Endopeptidase activity of wtLCA (●) and tLCA () at 25 °C and fLCA () and tLCA () at 37 °C. (**b**) Comparison of the percent cleavage of SNAP-25 by 50 nM fLCA (■) and tLCA () in M17 neuroblastoma cells after 48 hrs. The percent cleavage was calculated from the densitometric analysis of the cleaved and uncleaved band of SNAP-25 in Western blot (inset). Error bars represent the standard deviation of triplicate samples.

The endopeptidase activity of fLCA and tLCA was examined using a peptide substrate (Nutide) at 25 °C and 37 °C and compared with the activity of its native substrate in M17 neuroblastoma cells. A peptide substrate offers insight into the availability of the active site, as this is the only region targeted by the peptide; full-length substrate offers insight into the global folding of the molecule. Cleavage of Nutide was over three-fold higher for both fLCA and tLCA at 37 °C compared to 25 °C (3.2- and 3.1-fold higher, respectively), after 30 min (Fig. 1a). This optimum activity at 37 °C has been observed previously and linked to the pre-imminent molten globule enzyme (PRIME) conformation for fLCA^5^. Despite the similar increase in activity at 37 °C compared to 25 °C, tLCA showed a lower final activity than fLCA at 25 °C and 37 °C (1.4% vs. 3.9% cleavage at 25 °C; 4.5% vs. 12% cleavage at 37 °C, seen in Table 1). Notably, the ratio of activity of tLCA (37 °C activity compared to 25 °C) is similar to the ratio of activity of fLCA, and the lower endopeptidase activity of tLCA indicates a structural and functional difference between the two proteins.

**Table 1:** Summary of activity of fLCA and tLCA with peptide and full-length substrates.

LCA	Temp. (°C)	Substrate	% cleavage	% activity compared to fLCA at 37 °C
fLCA	25	peptide	3.9% ± 0.3%	33%
fLCA	37	peptide	12% ± 1.8%	100%
tLCA	25	peptide	1.4% ± 0.1%	12%
tLCA	37	peptide	4.5% ± 0.3%	38%
fLCA	37	full-length	38.9% ± 1.1%	100%
tLCA	37	full-length	19.6% ± 1.4%	50%

Shown are the % cleavage of substrates after 30 min (peptide) or 48 hrs (full-length in M17 neuroblastoma cells), each performed in at least triplicate.

When observed at 10 min, tLCA also showed a slower initial rate of activity (activity curve data shown as Fig. 1a). While fLCA at 10 min showed 32% (25 °C) and 42% (37 °C) of the final activity, tLCA at 10 min only showed 18% (25 °C) and 35% (37 °C).

SNAP-25 is an intracellular native substrate for LCA. To compare the activity of fLCA and tLCA with respect to their native substrate intracellularly, M17 neuroblastoma cells were used. Even with the native substrate, tLCA again showed significantly less activity than fLCA (19.6% cleaved SNAP-25 compared to 38.9%), showing the tLCA only to be 50% active compared to fLCA (Fig. 1b). The relative activity of tLCA with intracellular SNAP-25 as substrate is 36% higher in comparison with Nutide as substrate (tLCA was 38% active with Nutide vs. 50% active with SNAP-25 compared to fLCA).

### Circular Dichroism of LCA

**Figure 2:**
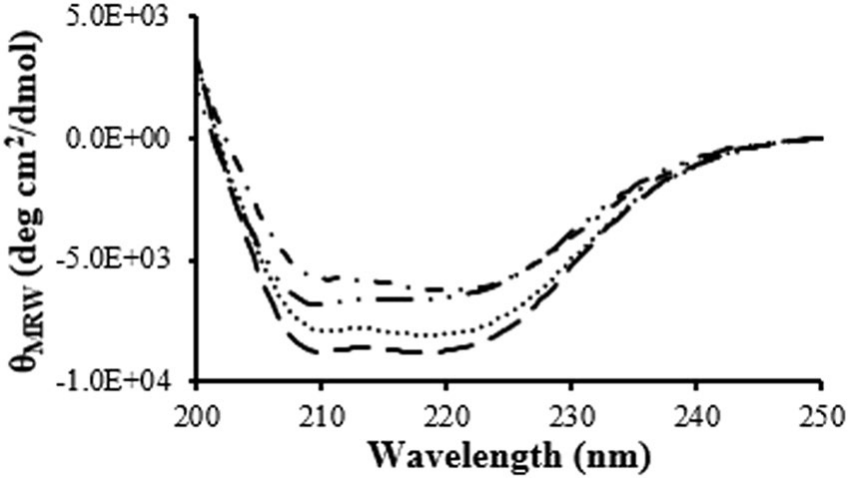
Far-UV CD spectra of fLCA and tLCA (dissolved in 10 mM sodium phosphate, pH 7.5 containing 150 mM NaCl) at 25 °C (fLCA;  tLCA: ) and 37 °C (fLCA: ; tLCA: • • •). Each spectrum is the average of at least 3 samples.

Far-UV and near-UV CD spectroscopy was used to monitor the secondary and tertiary structure of fLCA and tLCA, respectively, at different temperatures. Both LCAs showed minima near 220 nm and 208 nm, indicating a mostly α-helical globular protein (Fig. 2). There were significant differences between the fLCA and tLCA far-UV CD spectra at both 25 °C and 37 °C in terms of ellipticity. At 25 °C, tLCA showed a significantly stronger signal at both minima (34% and 29% higher at 222 nm and 208 nm, respectively), suggesting more α-helical content in tLCA.

Upon heating to 37 °C, the LCAs maintained significantly different far-UV CD spectra. The tLCA remained largely unchanged, decreasing only 12% and 8% ellipticity at 208 nm and 222 nm, respectively, with only a 5% increase to the θ_222_/θ_208_ ratio. On the other hand, fLCA maintained nearly the same ellipticity at 222 nm (3% decrease) yet decreased 19% ellipticity at 208 nm, indicating a loss of α-helical content. This change increased the θ_222_/θ_208_ ratio of fLCA by 20% at 37 °C.

**Figure 3:**
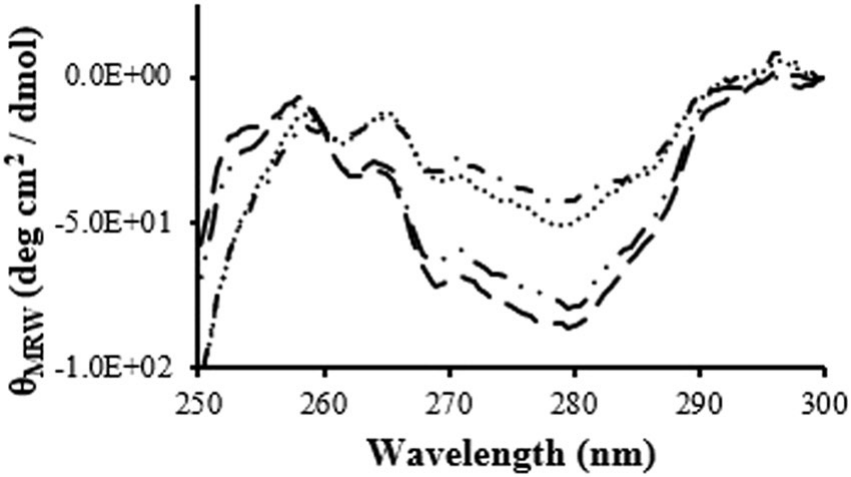
Near-UV CD spectra of fLCA and tLCA (dissolved in 10 mM sodium phosphate, pH 7.5 containing 150 mM NaCl) at 25 °C (fLCA: ; tLCA: ) and 37 °C (fLCA: ; tLCA: • • •). Each spectrum is the average of at least 3 samples.

The near-UV CD spectra of fLCA and tLCA were similar in shape at 25 °C, showing broad minima at 280 nm (asymmetry around Tyr) and minima at 269 nm and 263 nm (asymmetry around Phe) (Fig. 3), however tLCA showed an 8% higher signal at 280 nm compared to fLCA. These similarly shaped spectra indicate a similar asymmetry around the Tyr and Phe residues, and the small decrease in signal observed with fLCA is despite fLCA containing 25 Tyr compared to tLCA with 23 Tyr, suggesting a more flexible tertiary folding.

Both fLCA and tLCA contain 2 Trp residues which did not produce a well-resolved signal in the near-UV CD spectra at 25 °C. Both spectra arguably showed a small shoulder around 286 nm which could be partially due to contribution of one or both Trp residues, but it has also been argued that neither Trp show a peak because of the difference in the microenvironment of both these residues. Trp43 exists in a conformationally symmetrical segment of the protein, whereas the microenvironment of Trp117 is comparatively more flexible region of the protein^6,21^.

Heating to 37 °C decreases the asymmetry around the Tyr and Phe residues for both fLCA and tLCA. The fLCA and tLCA spectra had less distinctive peaks than those seen at 25 °C, however retains some definitive negative peaks at 269 nm and 263 nm with a broad negative area around 280 nm. Both LCAs show an increase in flexibility as evidenced by the general loss of signal, yet fLCA showed a decreased signal at 280 nm compared to tLCA at the same temperature (19% lower than tLCA), again suggesting a more flexible packing of fLCA in comparison to tLCA at 37 °C (Fig. 3).

### Steady state fluorescence and tryptophan fluorescence lifetime

The emission wavelength (λ_em_) of the LCA Trp residues was observed by selectively exciting the residues with a 295 nm excitation wavelength (λ_ex_). Each LCA contains two Trp residues (W43 and W117). The fLCA λ_em_ was 323 nm (similar to reported data by Kumar *et al*. (324 nm)^6^ and Ahmed *et al*. (322 nm)^22^), while the tLCA λ_em_ was 315 nm (Fig. S2). The blue-shifted λ_em_ of tLCA indicates the microenvironment around the two Trp residues being more hydrophobic than in fLCA and is towards the lower end of the typical emission spectra of buried Trp; however, a λ_em_ in this range was reported for LCB previously^6^. One factor in this difference is the solvent exposure of the Trp residues: fLCA Trp are more solvent exposed (red-shifted) which could be due to a more flexible tertiary structure coupled with less secondary structure than tLCA, as observed in far- and near-UV CD, allowing for water molecules to penetrate the enzyme; conversely, tLCA is more compact in nature due to more secondary structure, as observed with far-UV CD, and maintains a more condensed hydrophobic core, shielding the Trp from interactions with the water.

To further examine the environment around the LCA Trp residues, the Trp fluorescence lifetime was monitored. Trp in fLCA showed a one component lifetime of 2.14 ± 0.05 ns, tLCA Trp showed a one component lifetime of 1.93 ± 0.03 ns. The longer lifetime observed in fLCA is characteristic of more conformational flexibility of the microenvironment around the Trp residue. Intrinsic quenching of the Trp residues is also more likely in tLCA with more secondary structure present. A more compact enzyme would bring potential quenching groups from neighboring residues in closer proximity which would explain the lower lifetime of tLCA Trp and the more flexible/less structured fLCA would have quenching groups further away from the Trp. Additionally, the shorter lifetime for tLCA could be due to higher exposure of Trp to the more polar environment, however, this is unlikely to be the case based on the increased folding and blue-shifted fluorescence λ_max_ observed for tLCA. The longer lifetime of fLCA, despite less secondary and tertiary structure suggests that Trp residues are protected from relatively polar environment, and the lower Trp fluorescence lifetime of tLCA is basically due to intrinsic quenching^18^, which gets relieved in a more flexible structure observed in fLCA.

### Urea denaturation

**Figure 4:**
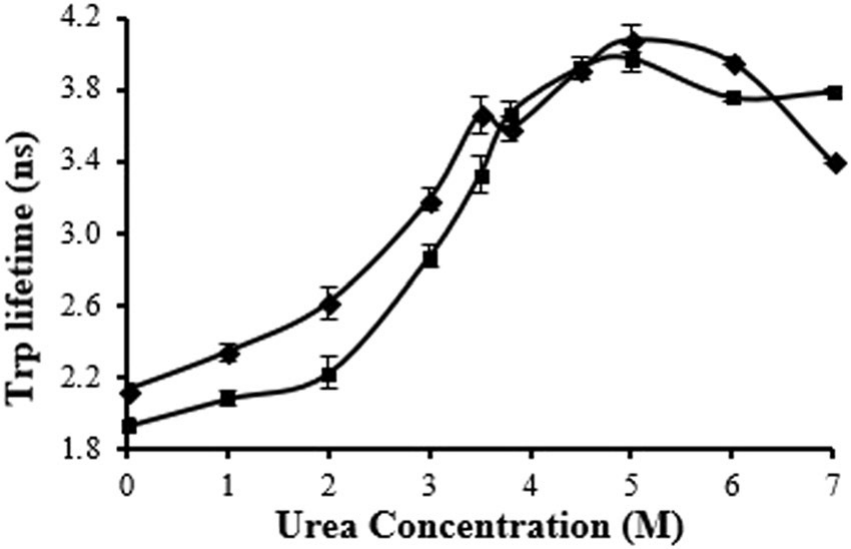
Comparison of urea denaturation of fLCA (closed diamond) and tLCA (open square) using Trp fluorescence lifetime. Error bars represent the standard deviation of at least 3 samples.

The fluorescence lifetime of Trp in fLCA and tLCA was monitored over a range of urea concentrations to monitor the packing of the tertiary structure (Fig. 4). Over the lower urea concentrations (0.0–2.0 M), the lifetime slowly increased and tLCA was more resilient to change than fLCA (22% increase for fLCA and 15% increase for tLCA between 0.0–2.0 M urea). This difference matches with the far-UV CD data observed in which tLCA contains more secondary structure than fLCA and would better resist changes to the overall structure, indicating a less accessible interior of tLCA.

Changes to each of the LCA Trp fluorescence lifetimes was most dynamic between 2.0–3.5 M urea. The fLCA lifetime increased nearly 40% over this range; while initially more resistant to change in lifetime at lower urea concentrations, tLCA increased 49% over the 2.0–3.5 M urea range (Fig. 4). At 3.75 M, both LCAs showed a different trend where the tLCA Trp fluorescence lifetime continued to increase after 3.5 M (13% increase), yet fLCA showed a small 2% decrease in the lifetime (reproducible, yet not statistically significant) between 3.5 M and 3.75 M urea. Interestingly, this 3.75 M urea concentration coincides with the I_1_ intermediate state observed previously^20^.

Both LCAs reached a global maximum for Trp fluorescence lifetime at 5.0 M urea yet with the lifetime of tLCA still slightly shorter than the fLCA. The transition between 3.75–5.0 M urea was more linear in the case of fLCA while tLCA began to level off at 4.5 M urea. As with 3.75 M urea, the 5.0 M urea was observed as an intermediate state (I_2_) in the study performed by Kumar *et al*.^20^.

Upon reaching the maximum Trp fluorescence lifetime at 5.0 M urea, both fLCA and tLCA showed a similar decrease in lifetime upon addition of 6.0 M urea (3% decrease for fLCA and 5% decrease for tLCA). A larger decrease in the fLCA Trp fluorescence lifetime was observed between 5.0–7.0 M urea (17%), however, the tLCA lifetime remains essentially unchanged between 6.0–7.0 M urea. The decrease in Trp fluorescence lifetime after 5.0 M urea can be explained by Trp becoming sequestered as hydrophobic regions condense to limit the exposure to the polar solvent as the protein unfolds and therefore limiting rotational flexibility of the residue. In the case of fLCA, this increased unfolding was shown by Kumar *et al*.^20^ where the fLCA increased from 40% random coil to 50% random coil between 5.0 M and 7.0 M urea. The θ_222_ showed a decrease between 5.0 M and 6.0 M urea, yet showed a much more substantial decrease between 6.0 M and 7.0 M urea which supports the larger decrease in Trp fluorescence lifetime between these two concentrations. Unlike the fLCA, tLCA did not show a steady decrease in Trp fluorescence lifetime past 5.0 M urea, instead leveling off after 6.0 M urea indicating the Trp residues remain shielded from the polar solvent. This basically is consistent with the hypothesis (i) intrinsic quenching of Trp fluorescence is the primary cause of the low Trp fluorescence lifetime, and (ii) structural response in the fLCA and tLCA is different due to differences in the rigidity of their structures.

### Binding of 1-Anilinonapthalene 8-Sulfonate (ANS)

ANS is a small molecule dye which is routinely used as a hydrophobic probe in fluorescence measurements in proteins and membranes^19,23^. The ANS dye has a high affinity for solvent-exposed hydrophobic clusters and proteins with a loosely packed core show increased binding to the dye^23,24^. To investigate the differential packing of fLCA, in comparison with tLCA, the fluorescence signal of ANS at 488 nm was monitored spectroscopically over temperature (25–55 °C).

**Figure 5:**
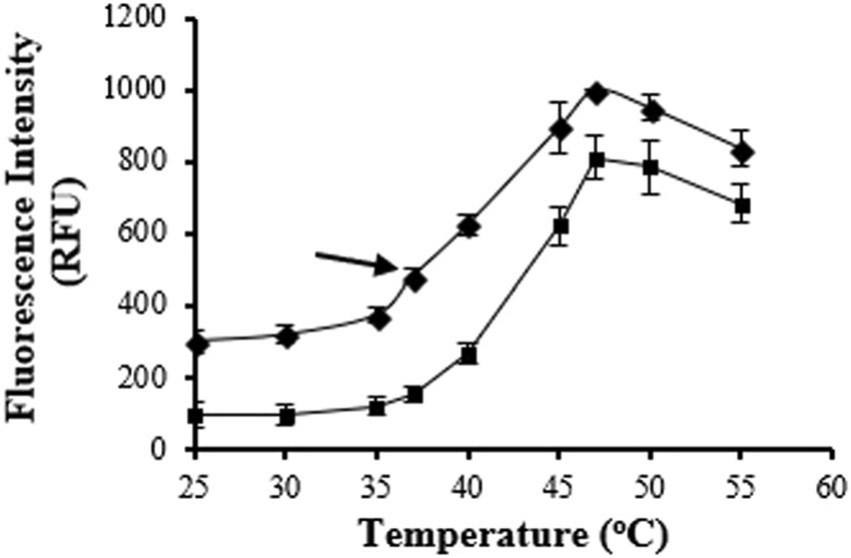
Comparison of ANS binding of fLCA (closed diamond) and tLCA (open square). A 70:1 ANS/protein ratio was used; excitation and emission wavelength of ANS was 370 nm and 488 nm, respectively. Error bars represent the standard deviation of at least 3 samples. The small bump at 37 °C indicated with an arrow was observed previously in fLCA and characterized as the PRIME conformation^5^.

Both fLCA and tLCA showed a maximum ANS binding at 47 °C (Fig. 4) and indicate their folding includes a molten globule intermediate. The fLCA also contained a second intermediate state at 37 °C (Fig. 5) which was described as the PRIME conformation previously^5^. This PRIME conformation is characterized by a loosened tertiary structure with intact secondary structure. Interestingly, tLCA does not show the small deviation in ANS binding at 37 °C which is a characteristic of the PRIME conformation.

At each monitored temperature, fLCA had a higher ANS fluorescence signal (Fig. S3) which would suggest a relatively looser packing of fLCA compared to tLCA. The fluorescence spectra of fLCA, particularly at 25 °C, is broader than tLCA (Fig. S3), typical of a more accessible microenvironment, and would support the looser packing of fLCA compared to tLCA.

### Circular dichroism of substrate bound LCA

The effect of substrate binding on each LCA was monitored using both far- and near-UV CD spectroscopy to distinguish any structural features that change upon binding to a full-length substrate. For this study, a cleavage-resistant form of the SN2 domain of SNAP-25 was used to interact with fLCA and tLCA. The far-UV CD spectra of SN2 (25 °C) closely resembled previously reported data of full-length SNAP-25, indicating a similar structure of the truncated SN2 version compared to the full-length substrate (Fig. S4)^25^, and was shown to be cleavage resistant at the concentrations used for CD measurements (Fig. S5).

**Figure 6:**
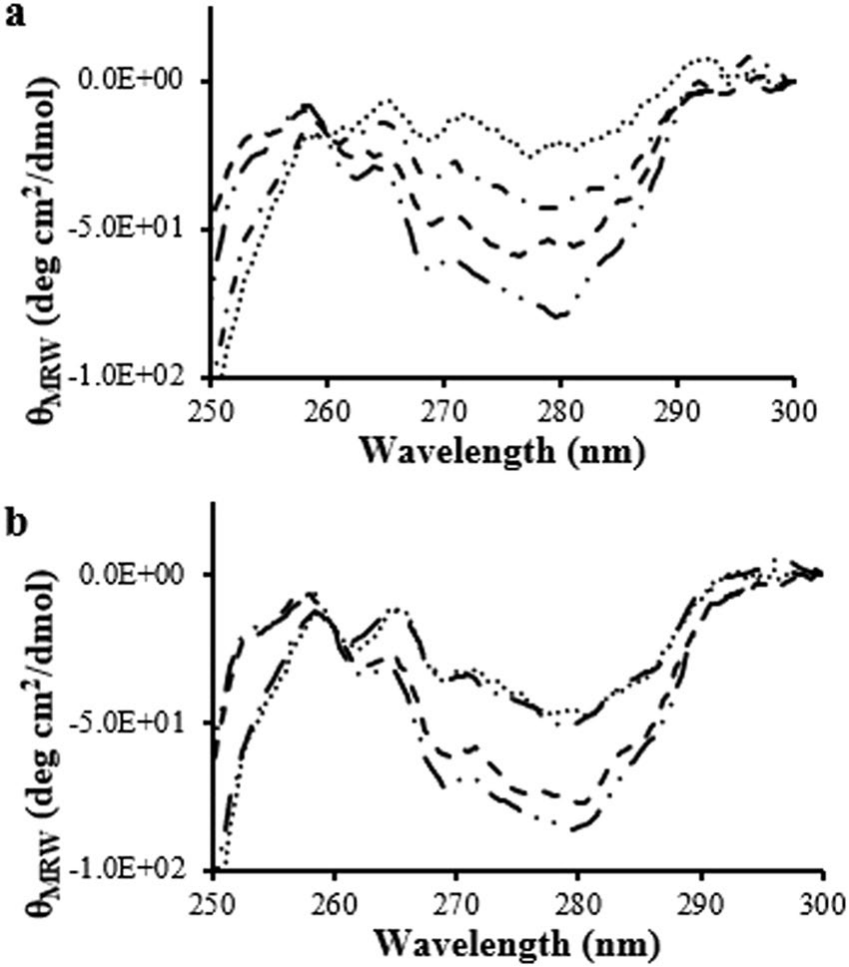
Near-UV CD data of LCA with and without 1:1 molar ratio SN2. (**a**) Shown are: bound () and unbound () fLCA at 25 °C and bound (• • •) and unbound () fLCA at 37 °C. Each spectrum is the average of at least 3 samples. (**b**) Shown are bound () and unbound () tLCA at 25 °C and bound (• • •) and unbound () tLCA at 37 °C. Each spectrum is the average of at least 3 samples.

The most apparent change to both LCA structures in the near-UV region was observed at 25 °C when bound with SN2, while only fLCA showed significant refolding at 37 °C (Fig. 6). Both fLCA and tLCA showed a decrease in ellipticity at 280 nm, 269 nm, and 263 nm after binding to SN2, with fLCA showing a consistently greater decrease in signal. At 280 nm, fLCA ellipticity decreased 25% while tLCA decreased 9%; fLCA showed a 19% decrease in ellipticity at 269 nm while tLCA showed a 16% decrease; fLCA showed an 18% decrease in ellipticity at 263 nm and tLCA showed a 11% decrease.

An overall loss in signal would indicate a more flexible environment around the aromatic residues. Based on the change in ellipticity at 25 °C, both LCAs appear to become more flexible, however, it appears that the fLCA undergoes a more significant loosening of the tertiary structure upon binding. This conformation change is supported by the change in peaks present in both fLCA and tLCA in the bound, but not unbound, near-UV CD spectra. Upon binding to SN2, both LCAs show negative peaks at 286 nm, 280 nm, 277 nm, 269 nm, and 263 nm (Fig. 6). The appearance of more resolved peaks at 286 nm and 277 nm in bound LCAs suggest a refolding of the tertiary structure upon binding. In the unbound state, both LCAs have a broad negative peak at 280 nm which could hide some of the finer details of the peak in that region and mask information about the 277 nm and 286 nm peak. Both LCAs showed a small shoulder near 286 nm, but the high signal observed at 280 nm may obscure this. Due to loss in the intensity of 280 nm peak (probably due to loss of asymmetry around tyrosine residues), and conformational changes around tryptophan residues, the peak at 286 nm was more clearly resolved.

Unbound LCAs, as mentioned earlier, were not able to resolve a clear Trp peak. While it is not clear which Trp residue is responsible for the 286 nm peak observed at 25 °C for bound LC, there is clear evidence that one, or both, Trp undergo a conformational change which increases the asymmetry around the residue(s).

The appearance of a 277 nm peak is observed in the bound form of both fLCA and tLCA and the signal was approximately the same intensity as observed at 280 nm. This peak was likely created by a change to the asymmetry around the Tyr residues. This small shift away from 280 nm could mean some of the Tyr residues remained in the same microenvironment as the unbound state and still showed a peak at 280 nm while other Tyr residues underwent a change in conformation, resulting in the rise of a peak at 277 nm.

At the optimally active temperature (37 °C), fLCA underwent substantial changes to the tertiary structure after binding to SN2 while tLCA remained largely unchanged. Bound fLCA had a 51% decrease in signal at 280 nm, whereas tLCA remained largely the same (Fig. 6). Bound tLCA at 37 °C showed a nearly identical spectrum to that seen in the unbound condition at 37 °C with the exception being the 280 nm peak observed in the unbound spectra becoming a split peak (280 nm and 277 nm) after binding to SN2 (Fig. 6). This considerable difference between the unbound and bound fLCA spectra at 37 °C suggests that at this temperature, the fLCA structure is significantly affected by the presence of substrate and is refolded into a more catalytically active conformation. On the other hand, tLCA is resistant to structural changes at 37 °C. Changes to the structure of SN2 upon binding are very unlikely to be the reason behind the spectral changes observed as SN2 only contains 1 Tyr and 1 Phe, both of which are in the His-tag region of SN2 that would not bind to the LCA. These observations could be significant when explaining the differences in catalysis rates of the LCAs.

**Figure 7:**
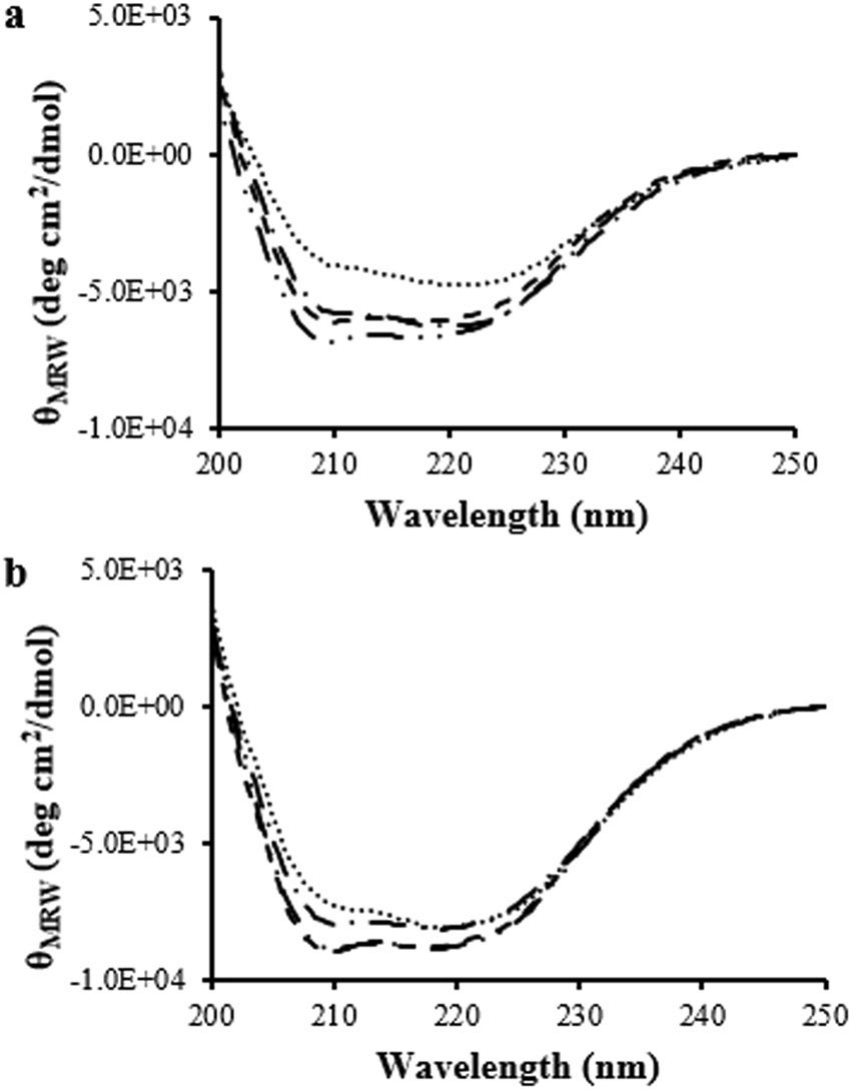
Far-UV CD data of LCA with and without 1:1 molar ratio SN2. (**a**) Shown are: bound () and unbound () fLCA at 25 °C and bound (• • •) and unbound () fLCA at 37 °C. Each spectrum is the average of at least 3 samples. (**b**) Shown are: bound () and unbound () tLCA at 25 °C and bound (• • •) and unbound () tLCA at 37 °C. Each spectrum is the average of at least 3 samples.

The changes to the LCA secondary structure upon binding to SN2 was also monitored using far-UV CD. Changes to the peaks at 222 nm and 208 nm were indicative of secondary structure changes. Bound fLCA and tLCA at 25 °C showed small decreases in ellipticity at 222 nm (8% and 2%, respectively), and maintained similar θ_222_/ θ_208_ ratios (Fig. 7). More significant changes to the spectra were observed at 37 °C for both LCAs. Bound tLCA did not show any change to the ellipticity at 222 nm upon binding, however, the ellipticity at 208 nm decreased 11% (12% increase in θ_222_/ θ_208_ ratio). Bound fLCA showed a decrease in ellipticity at both 222 nm and 208 nm (23% and 32%, respectively; 14% increase in θ_222_/ θ_208_ ratio). Changes to the secondary structure of SN2 upon binding to LCA may occur and subtracting the free SN2 spectra from the LCA/SN2 complex spectra may not account for this. However, a composite spectrum of LCA + SN2 was calculated and compared to the LCA/SN2 complex spectra and the observations made were consistent (data not shown). In summary, binding of substrate affects secondary and tertiary structure of fLCA more than tLCA.

### Binding kinetics of fLCA and tLCA to SN2

SPR-based technology was used to characterize fLCA and tLCA binding affinity to SN2. The 1:1 binding model is a better fit with lower chi^2^ value compared to a bivalent binding and a heterogeneous ligand binding model in the BIAevaluation software, therefore the binding parameters between LCA and SN2 were calculated using the 1:1 binding model. The equilibrium binding constants, association, and dissociation rates were obtained by applying the 1:1 binding model from the series of several concentrations of analytes (Fig. S6). The results clearly suggested that the fLCA showed a fast association phase accompanied by a slower dissociation phase compared to that of tLCA. The binding constants (K_D_) of fLCA and tLCA were 0.027 ± 0.002 nM and 5.7 ± 1.8 nM, respectively (Table 2). The fLCA showed greater than 200× higher affinity for SN2 compared to tLCA, which is largely due to a significantly faster association rate constant (~60× of tLCA), combined with a slower dissociation rate constant (~one third of tLCA). These differences between fLCA and tLCA suggest fLCA can bind to its substrate more effectively (faster binding and slower dissociation) compared to tLCA.

**Table 2:** Calculation of binding kinetics of fLCA and tLCA to the immobilized SN2. The SN2 was immobilized on a CM3 chip and binding kinetics were calculated using a 1:1 binding kinetics model.

	k_a_ (M^−1^ s^−1^)	k_d_ (s^−1^)	K_D_ (nM)	Chi² (RU²)
fLCA/SN2	6.20 ± 0.02 × 10^6^	1.7 ± 0.2 × 10^–4^	0.027 ± 0.002	0.89
tLCA/SN2	1.03 ± 0.03 × 10^5^	5.79 ± 0.08 × 10–4	5.7 ± 1.8	0.185

Standard deviations were calculated from at least 3 samples.

## Discussion

Botulinum neurotoxins are highly evolved proteins producing some of the highest biologically effective characteristics, as expressed in terms of their biological toxicity. Produced by an over 2-billion-year-old anaerobic bacteria, the toxin has evolved to be an extremely effective biological molecule with at least the following three characteristics: (i) Targeting the most evolved animal system, namely the nervous system, which controls the functioning of the entire animal body; (ii) Very long lasting protein molecule inside a foreign cell; (iii) The toxin exerts its function through the blockade of neurotransmitter release at the nerve-muscle junctions. The molecular basis of these three features can reveal the mystery of not only botulinum neurotoxins, which are listed as Class A biothreat agents while also being used as one of the most versatile biotherapeutic against over 800 human disorders, but also will help understand the evolution of effective biomolecules, in general.

The light chain of botulinum neurotoxins plays the key role in its toxicity and therapeutic activity through its highly selective endopeptidase activity towards components of the SNARE complex in neurons. BoNT/A is the most toxic and most effective drug amongst the serotypes of BoNTs^26–29^. An accurate structure of the type A light chain is critical for molecular involved in the action of BoNT, and for the design of inhibitors as antidotes against botulism. There has been some debate about post-translational modifications of the full-length LCA. The BoNT/A gene codes for 448 residues, however one report cites the C-terminus at residue 437^30^, another cites it as 444^31^, and the crystal structure of the BoNT/A toxin (3BTA) potentially has 448 residues^32^. PDB validation of 3BTA does not have coordinates for residues 432–449, however the region could be flexible with residue coordinates not able to be established, and not a lack of residues present.

It remains unclear how the LCA exists within the *C. botulinum* bacteria, and eventually neurons, and what effect the processing and handling in the isolation of LCA has on the post-translational modification. Numerous reports have utilized full-length LCA with no post-translational modifications to identify structural and functional aspects of the catalytic domain^5–7,17,18,20,22,33–39^. Based on the detailed analysis of Dekleva and DasGupta^31^, the full-length LCA being used by various researchers is not in fact full-length LCA, but a full-length LCA + 4 residues. Mizanur *et al*.^7^ have observed that the minimum length of the LCA is 444 for optimum enzymatic activity. Nevertheless, the full-length LCA as used in this study, and numerous other studies, demonstrates the highest enzymatic activity, especially in contrast to the tLCA commonly used for structural and inhibitor development studies^7–16^. This report aims not to suggest or imply the native length of LCA, but to offer insight into the structural and functional differences between two of the most commonly used variants of LCA in the literature. Historically, a truncated form of the LCA has been widely used for biochemical studies, in part due to its solubility and stability. While truncated LCAs remain popular and provide benefits, such as increased solubility and yield, many of the variants have been shown to be less catalytically active^7,13,16–18,40^. With tLCAs being less than fully active, yet being highly soluble and stable, this provides an opportunity to examine the structure function relationship of this high impact molecule.

This decreased catalytic activity in the truncated LCAs is likely due to structural differences of the protein which would alter the way, for example, inhibitors bind and alter enzymatic activity^4–46^. This is likely because of the structural and enzymatic differences between the truncated LCAs used *in vitro* compared to the full-length LCA present during botulinum intoxication. For the first time, this study has characterized significant structural and functional differences between fLCA and tLCA in both the unbound and substrate-bound states using a series of biological and biophysical techniques, such as far- and near-UV CD, enzymatic activity, SPR, and fluorescence. Understanding the structural and enzymatic differences between fLCA and tLCA can help improve inhibitor discovery and design.

In both the unbound and substrate-bound state, tLCA maintains a more compact conformation than fLCA. Differences between the far-UV CD spectra have been reported previously^18^, yet, these differences were never further evaluated. The differences between the far-UV and near-UV CD signals, tryptophan fluorescence intensity and lifetime, and ANS binding of fLCA and tLCA point to an increased secondary structure with a more compact tertiary structure organization in tLCA, particularly at 37 °C; this would support the data reported earlier in which tLCA showed a higher T_M_ than fLCA, indicative of a more compact structure^7^.

The implications of the more compact nature of tLCA, and the apparent lack of a PRIME conformation as described previously for fLCA^5^, may play a role in the decreased enzymatic activity with both peptide and full-length substrates. The optimum activity of both LCAs was observed at 37 °C, yet tLCA has consistently been observed to have less activity than fLCA^8–10,32^. The more compact secondary and tertiary structure of tLCA at both 25 °C and 37 °C could provide steric hindrances and occlude the active site from optimum interaction with a peptide substrate, or possibly causing a buildup of cleaved product in the active site^9^, whereas a more loosely packed fLCA more readily facilitates substrate interaction and dissociation at the active site. The active site of LCA-424 has been previously shown to be a flexible region^19^, however, this flexibility in the active site region could be further increased through the fluctuations of the unstructured C-terminus inducing a global loosening of the overall structure.

Binding of the SN2 domain of SNAP-25 with the LCAs diminishes the structural rigidity of the enzyme at 25 °C and 37 °C, as indicated by both far-UV and near-UV CD spectra (Figs 6 and 7), however, tLCA is more resistant to structural refolding. This increase in flexibility at the secondary and/or tertiary folding level after binding to SN2 is counterintuitive; binding with substrate would typically limit the flexibility of a protein or increase the amount of secondary or tertiary structure present in the protein and therefore increase the CD ellipticity signal, especially with such an extensive number of contact points spanning large stretches of the protein as is the case between SN2 and LCA. This unique behavior of LCA with its substrate is further evidence of a complex solution structure with notable differences between the available crystal structures.

Flexibility differences between fLCA and tLCA could initially play an important role in the unbound state; increased flexibility and surface area may explain the increased substrate binding demonstrated by SPR, perhaps through one of the two mechanisms: (i) High flexibility creates dynamic oscillations in different domains which resonate with a compatible dynamic within the largely unfolded structure of the SN2 domain of the SNAP-25. Increased dynamics has been predicted in the PRIME conformation of the LCA^6^; (ii) A fly-casting mechanism similar to that proposed by Shoemaker *et al*.^47^, a novel concept for LCA and as exhibited in Fig. 7, showing the initial binding step of LCA with SNAP-25.

The C-terminus appears to play a critical role in maintaining a flexible fLCA structure, via disruption of the intramolecular forces (H-bonds, salt bridges, hydrophobic interactions) at a specific frequency and in a selective sequence, leading to a more effective binding with the substrate. The 24-residue C-terminus, previously predicted to be flexible in nature^7^, acts as the handle of the fly cast whose truncation in tLCA leads to the collapse of the flexible folding forcing the protein towards a more compact structure. The C-terminus had previously been suggested to play a role in removing the product from the active site^7^; our proposed role of the C-terminus does not necessarily support or disagree with this model as the focus here was on the initial binding step rather than the catalysis step.

**Figure 8:**
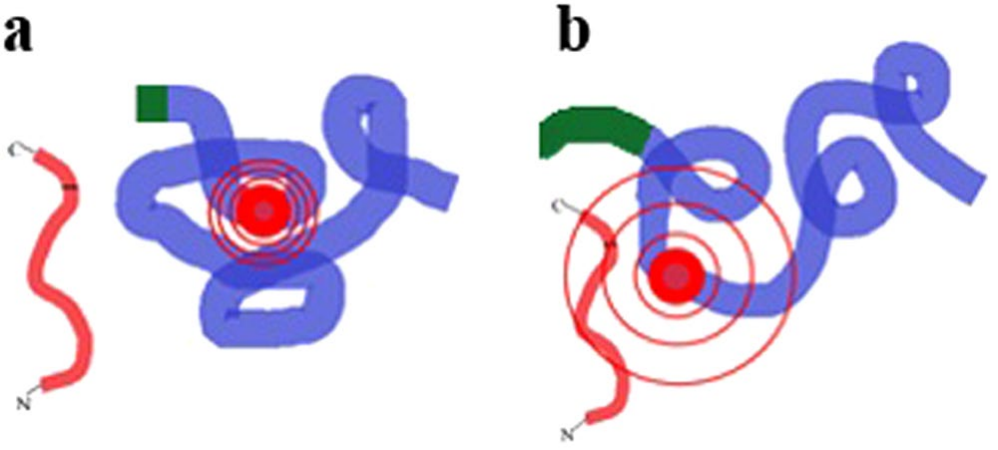
An illustration of the novel LCA “fly-casting” mechanism. The C-terminus of LCA () helps maintain a more flexible global conformation in fLCA (). The tLCA (**a**) has a truncated C-terminus which limits the global flexibility of the LCA while the more extended C-terminus of fLCA (**b**) is responsible for a looser structure with more surface area exposed. When LCA approaches the binding site of SNAP-25 (C-  -N), the LCA in a less flexible conformation (**a**) offers a smaller capture radius of the α-exosite () compared to the more flexible structure (**b**). The initial contacts made by the flexible conformation are weak, but they allow the protein to “reel” itself into the binding site to complete the process. In contrast to the suggestion by Shoemaker *et al*.^47^, our results suggest the binding of substrate, in fact, makes the enzyme structure more open and flexible.

At the molecular level, the SNAP-25 initially binds at the α-exosite and then binds to multiple contact points, like a zipper, as it wraps around the LCA^48^. The differences in LCA flexibility coupled with the consistent differences in endopeptidase activity between fLCA and tLCA suggest the α-exosite is in a more suitable binding/capturing conformation in fLCA. The α-exosite of fLCA with expanded structure would have a larger capture radius, while tLCA would have a smaller capture radius to form the initial contacts with SNAP-25 (Fig. 8). Upon binding, our data suggests the SNAP-25 refolds the LCA towards an even more flexible conformation, in contrast to the original fly-cast mechanism proposed^47^. This refolding of LCA upon α-exosite binding has been discussed previously in which it was shown to increase the catalytic rate of a peptide substrate^10^, likely by increasing the exposure of the active site.

The rapid binding of the SN2 domain to fLCA coupled with slower dissociation upon binding, determined using SPR, supports this fly-casting mechanism (Fig. 8). The flexible structure of fLCA plays a critical role for capturing its substrate and facilitates the binding with slower dissociation than that observed with tLCA. This possible mechanism may be the key to understanding the decreased endopeptidase activity of tLCA compared to fLCA and could potentially be exploited in the search for effective inhibitors. Inhibitors which target the C-terminus, or inhibit the mobility, may be more effective in decreasing the activity of LCA.

Functionally disordered proteins have become accepted relatively recently as more protein structures are solved^49,50^. These proteins which contain disordered regions, domains, or are entirely disordered have been observed to play critical roles in overcoming steric constraints of binding^51,52^ and cell signaling pathways^53–55^; disorder has also been predicted in several toxins, such as botulinum neurotoxin, tetanus toxin, anthrax lethal factor, or diphtheria^56^. The flexibility achieved after LCA binds to its substrate likely is composed of some disordered regions throughout the protein. This uncommon strategy of a protein/substrate complex becoming more flexible upon association demonstrates how unique the botulinum neurotoxin light chain truly is and how vital a better understanding of the solution structure will be for researchers.

The differences noted in this study offer valuable insight into (i) a better understanding of the LCA structure in a solution state as well as its interaction with the native substrate, (ii) a unique observation that substrate binding is more effective with a more flexible enzyme structure, leading to further loosening of the structure to accompany higher activity. These insights will be helpful in developing an improved method for *in vitro* inhibitor discovery which can be better translated into an antidote under the *in vivo* conditions. More importantly, the results suggest that an evolutionary trait of flexible structure with specific dynamic motion may explain the molecular and physiological behavior of the most potent molecule that survives in a foreign cell for months to be effective in its biological function.

## Materials and Methods

### Purification of fLCA, tLCA, and SN2

The purification of LCAs (fLCA: BL21 STAR (DE3) cells, New England Biolabs, Ipswich, MA, ampicillin resistance; tLCA: BL-21(DE3)-RIL cells, kanamycin sulfate resistance, kindly provided by Dr. Subramanyam Swaminathan) was adapted from previously reported studies^10,30^. Each LCA is a recombinant version of the Hall A1 strain. LCAs were grown in ~50 mL 2YT media +100 μg/mL antibiotics (ampicillin for fLCA; kanamycin sulfate for tLCA) overnight at 37 °C and then ~15 mL of the culture was used to inoculate 1 L 2YT media +100 μg/mL antibiotics and grown at 37 °C, with 250 rpm shaking, (Innova 4000 Incubator Shaker; New Brunswick Scientific, Edison, NJ) until the OD_600_ was 0.6–0.8. The LCAs were induced with 1 mM IPTG (isopropyl β-D-1-thiogalactopyranoside) for 16 hrs with 75–100 rpm shaking at 25 °C (fLCA) or 18–22 °C (tLCA). Cells were harvested by centrifugation at 7,000 rpm for 10 min at 4 °C and frozen at −20 °C.

The cells were resuspended in ~50 mL lysis buffer/L pelleted cells. Lysis buffer contained 10 mM sodium phosphate, 150 mM NaCl, 1 mM PMSF (phenylmethanesulfonyl fluoride), and 5 mM benzamidine, pH 7.5. Cells were ruptured by pulse sonication for 3 × 1.5 min at 12 W on ice then centrifuged at 11,000 rpm for 20 min at 4 °C to remove cell debris. The supernatant was passed through a HisPur Ni-NTA column (3 mL bed volume; ThermoFisher Scientific, Waltham, MA) which had been equilibrated with lysis buffer minus the protease inhibitors (sample buffer). The column was washed with 15 mL sample buffer, 15 mL of 20 mM imidazole dissolved in sample buffer, then eluted in 200 mM imidazole in sample buffer. Eluted fractions were dialyzed against sample buffer to remove the imidazole. Concentration of the LCA was measured by UV/Vis spectroscopy using the ε_280_ for fLCA (0.83 mg^−1^ mL cm^−1^) or tLCA (0.91 mg^−1^ mL cm^−1^, converted from molar extinction coefficient) reported previously^13,57^.

A plasmid of cleavage-resistant recombinant SN2 domain of SNAP-25 was synthesized and purified at Blue Heron (Bothell, WA) in a pEX-N-His vector with a T7 promoter and transformed into BL21 STAR (DE3) cells (ThermoFisher Scientific). Cleavage-resistant recombinant SN2 is a His-tagged truncated version of human SNAP-25 and consists of amino acid residues 136–206 of the protein (underlined below) with a mutation, R198A, to eliminate the cleavage of the substrate (cleavage site bolded):

MHHHHHHENLYFQGAIARVTNDARENEMDENLEQVSGIIGNLRHMALDMGNEIDTQNRQIDRIMEKADSNKTRIDEAN**QA**ATKMLGSG.

R198A SNAP-25 has been previously shown to be resistant to cleavage using fLCA^49,50^; this resistance to LCA cleavage was confirmed by incubating each LCA with the SN2 using conditions from the CD experiments and visualizing using SDS-PAGE.

Recombinant SN2 was grown and purified using the same conditions as fLCA with a 12 hr induction time. Without a published extinction coefficient available, the Whitaker Method was utilized to determine protein concentration^58^:

1 $$C(\frac{mg}{mL})=\frac{{A}_{235}-{A}_{280}}{2.51}$$

The molecular weight of the SN2 was calculated to be 10.04 kDa. The far-UV CD spectra was recorded at 25 °C and compared favorably to a previously reported spectrum of full-length SNAP-25^25^. To confirm the SN2 was cleavage resistant, SN2 was incubated with each LCA at the concentrations used for CD measurements. The samples were incubated at 25 °C for 15 min, followed by an incubation at 37 °C for 15 min to replicate the incubation conditions of the CD measurements. Reactions were stopped using SDS sample buffer and run on a 4–20% SDS-PAGE gel and visualized.

### LCA endopeptidase activity using Nutide

The endopeptidase of fLCA (full-length LCA, residues 1–448) and tLCA (truncated LCA, residues 1–424) was examined using Nutide (FITC(bA)T(dR)IDQANQRAT(K/DABCYL)(Nle)-amide; 2224 g/mol, New England Biolabs), a synthetic FRET based peptide containing the BoNT/A native cleavage site (underlined in the sequence above)^48^. Each LCA was preincubated (50 nM LCA dissolved 20 mM HEPES, pH 7.5) at the designated temperature (25 °C or 37 °C) for 15 min followed by the addition of 4.0 µM Nutide. Endpoint readings were read every 5 min for 60 min total on a SpectraMax M5 Plate Reader (Molecular Devices, San Jose, CA) using an excitation and emission wavelength of 490 nm and 523 nm, respectively, with a cutoff filter set at 495 nm.

The increase in fluorescence was calculated (in relative fluorescence units (RFU)) by subtracting the blank from the readings at each time point of the reaction. The % cleavage for each reaction was calculated by comparing to the signal of fully cleaved 4.0 µM Nutide; 4.0 µM Nutide was incubated with an excess amount of fLCA for 4 hrs to determine the maximum signal obtained for designated concentration.

### LCA endopeptidase activity in M17 neuroblastoma cells

Determination of the endopeptidase activity of the fLCA and tLCA was examined in M17 neuroblastoma cells. M17 neuroblastoma cells were grown in DMEM media containing 10% FBS (fetal bovine serum, ATCC, Manassas, VA) and 1% pen-strep antibiotic. Cells were seeded in 12 well plates with 2.5 × 10^5^ cells per well and maintained in a humidified 5% CO_2_ atmosphere at 37 °C. Media was changed the following day and the cells were allowed to grow to ~80% confluence. The endopeptidase activity of fLCA and tLCA with endogenous SNAP-25 in the cytosol of neuroblastoma cells was studied by delivering the LCAs inside the cytosol. Cytosolic delivery of 50 nM fLCA or tLCA was achieved using Lipofectamine 2000 (Life Technologies, Grand Island, NY); control cells received no LCA treatment^59,60^.

Experimental wells were harvested by trypsinization after 48 hrs. Cells were then lysed with a mammalian protein extraction reagent, M-PER (Piercenet, Rockford, IL), boiled for 5 min in Laemmli sample buffer, and then loaded on a 12% Mini Protean TGX stain free gel (Bio-Rad Laboratories, Hercules, CA). Protein samples were separated by sodium dodecyl sulfate polyacrylamide gel electrophoresis (SDS-PAGE) (Bio-Rad Laboratories, Hercules, CA) followed by transferring bands to a PVDF membrane. The blot was incubated with 5% non-fat dry milk in TBST for 1 hr at room temperature, followed by incubation with rabbit anti-SNAP-25 (1:2,500) antibody (Sigma-Aldrich, St. Louis, MO) for 1 hr at room temperature. Following the primary antibody treatment, the blot was incubated with rat-anti-rabbit (1:30,000) antibody (Sigma-Aldrich, St. Louis, MO) linked with alkaline phosphatase enzyme. Blots were visualized with BCIP (5-Bromo-4-Chloro-Indolyl-Phosphatase) development solution (Sigma-Aldrich, St. Louis, MO). Signals were scanned by a Bio-Rad imager and analyzed using the Bio-Rad image lab software.

### Far- and near-uv circular dichroism of LCA

Circular dichroism (CD) spectra of each LCA in an unbound state and substrate-bound state. and free SN2 were acquired with a Jasco J-810 spectropolarimeter. Far-UV CD spectra (200–250 nm) were recorded in 1.0 mm path-length cuvettes containing either 0.20 mg/mL LCA or a 1:1 mixture of LCA to SN2 in 10 mM sodium phosphate (pH 7.5) containing 150 mM NaCl. Near-UV CD spectra (250–300 nm) were recorded in a 10 mm cuvette using the same concentration of proteins in the above buffer. Each spectrum was recorded at 25 °C or 37 °C after a 5 min equilibration period using a 20 nm/min scanning speed, a response time of 8 sec, a 1 nm bandwidth, and a spectral resolution of 0.5 nm, and each spectrum was the average of three scans. Spectra were corrected for the buffer spectrum (or SN2 contribution), converted to mean residue weight ellipticity, and baseline corrected to adjust for temperature effects.

### Steady state fluorescence and fluorescence lifetime of tryptophan residues in LCA

Fluorescence spectra were recorded on an ISS K2 multifrequency phase fluorometer (Champaign, IL) using 0.05 mg/mL LCA samples. LCAs were excited at λ = 295 nm and emission spectra were recorded from 305–400 nm with excitation and emission slit widths fixed at 4 and 8 nm, respectively. Spectra were normalized at 310 nm.

Fluorescence lifetimes of LCAs were carried out on the instrument used above using an excitation wavelength of 300 nm provided by a LED light source and all measurements were recorded at 20 °C. Lifetime data was recorded using 15 modulation frequencies which were logarithmically spaced from 2 to 200 MHz. A 335 nm high-pass filter was used to remove scattered emission light and measurements were made with the emission polarizer set at the magic angle of 54.7°. A reference of *p*-terphenyl in absolute ethanol (τ = 1.05 ns) was used and LCA samples used were 0.1–0.2 mg/mL. The data was analyzed using the Vinci software provided by ISS, Inc. The fluorescence lifetime of Trp was modeled with one Lorentzian distribution with standard deviation of the phase and modulation ratio set at 0.20° and 0.008, respectively. The fraction, lifetime, and width of the Lorentzian distribution were the floating parameters.

Chemical unfolding of the LCAs was monitored using the tryptophan fluorescence lifetime over range of urea concentrations (0–7 M urea). Samples of LCA (0.1–0.2 mg/mL final concentration) were added to different urea concentrations and allowed to incubate at room temperature for 15 min before the lifetime was observed. A shorter incubation time was chosen to minimize aggregation of LCA which would interfere with the measurements; several samples of LCA were incubated for up to 2 hrs with no significant difference in lifetime (data not shown).

### Binding of 1-anilinonaphthalene 8-sulfonate (ANS)

The interaction of the fluorescent dye ANS with both fLCA and tLCA was monitored by measuring the fluorescence of the dye as a function of temperature (25–55 °C) when bound to each LC. Fluorescence measurements were recorded on an ISS K2 fluorometer using previously established procedures^42^. A dye to protein ratio of 70:1 (in μM) was used in a 10 mM sodium phosphate buffer, pH 7.4, containing 50 mM sodium chloride, and 1 mM DTT. The excitation wavelength was fixed at 370 nm and the emission spectra were recorded between 450 nm and 520 nm with excitation and emission slit widths fixed at 4 nm and 8 nm, respectively. The fluorescence intensity was corrected for the blank signal of ANS in the absence of protein. Intensity measurements were normalized in which the intensity measured for fLCA at 47 °C was set to 1000 RFU.

### Binding affinity of fLCA and tLCA to SN2 using surface plasmon resonance

Binding affinity between fLCA or tLCA with SN2 was studied using surface plasmon resonance (SPR) technology with a Biacore T100 (GE Healthcare Life Science, Piscataway, NJ). SN2 was covalently immobilized on a CM3 chip (GE Healthcare Life Sciences, Piscataway, NJ) through amine coupling following the instructions from the manufacturer. In brief, a CM3 sensor chip primed with PBS pH 7.4 followed by 50 mM sodium hydroxide solution prior to immobilization of SN2 on to the chip surface. The chip was subsequently activated with 0.2 M EDC (1-Ethyl-3-(3-dimethylaminopropyl)-carbodiimide), 0.05 M NHS (N-hydroxysuccinimide) solution. SN2 (3 µg/ml) was flowed over the CM3 chip for 5 min at a flow rate of 10 µL/min running buffer (PBS buffer (1×) containing 0.1% P-20) and obtained 392.0 RU. The chip was deactivated with 1 M ethanolamine (pH 8.5) and primed twice with binding buffer.

Freshly purified LCAs were used and diluted to the designated concentrations used in the experiment (0, 2.5, 5, 10, 20, 40, 60, 89, 133, 200, and 300 nM). The 1 nM and 40 nM were repeated to check the accuracy of the fLCA and tLCA samples, respectively.

Binding analysis was performed by maintaining the chip at 25 °C and analytes (LCAs) were injected at 30 μL/min for 2 min followed by a dissociation phase of 5 min. Each analyte injection was followed by 30 sec pulses of regeneration buffer (50 mM NaOH) and running buffer, respectively. Binding sensorgrams were further analyzed using the BIAEvaluation software using 1:1 binding kinetics model.

### Data Availability

The data generated in this study is available upon request to the corresponding author.

## Electronic supplementary material

Supplementary Information

## References

[1] Schiavo G, Matteoli M, Montecucco C (2010). Neurotoxins affecting neuroexocytosis. Physiol. Rev..

[2] Montecucco C, Rossetto O, Schiavo G (2004). Presynaptic receptor arrays for clostridial neurotoxins. Trends Microbiol..

[3] Rossetto O (2000). Bacterial toxins with intracellular protease activity. Clin. Chim. Acta.

[4] Kumar, R. & Singh, B. R. Evolution of Toxin, in *Protein Toxins in Modeling Biochemistry* (Kumar, R. and Singh B. R. Eds) 1^st^ ed., pp 113–134, Springer Briefs in Biochemistry and Molecular Biology, ISBN 978-3-319-43538-1 (2016).

[5] Kukreja RV, Singh BR (2005). Biologically active novel conformation state of botulinum, the most poisonous poison. J. Biol. Chem..

[6] Kumar R, Kukreja RV, Cai S, Singh BR (2014). Differential role of molten globule and protein folding in distinguishing unique features of botulinum neurotoxin. Biochim. Biophys. Acta.

[7] Mizanur RM (2013). The C terminus of the catalytic domain of type A botulinum neurotoxin may facilitate product release from the active site. J. Biol. Chem..

[8] Minnow YVT, Goldberg R, Tummalapalli SR, Rotella DP, Goodey NM (2017). Mechanism of inhibition of botulinum neurotoxin type A light chain by two quinolinol compounds. Arch. Biochem. Biophys..

[9] Guo J (2015). Substrate-based inhibitors exhibiting excellent protective and therapeutic effects against Botulinum Neurotoxin A intoxication. Sci. Rep..

[10] Xue S, Javor S, Hixon MS, Janda KD (2014). Probing BoNT/A protease exosites: Implications for inhibitor design and light chain longevity. Biochemistry.

[11] Stura EA (2012). Structural framework for covalent inhibition of Clostridium botulinum neurotoxin A by targeting Cys^165^. J. Biol. Chem..

[12] Cardinale SC (2011). Novel benzimidazole inhibitors of botulinum neurotoxin/A display enzyme and cell-based potency. Botulinum J..

[13] Kumaran D, Rawat R, Ahmed SA, Swaminathan S (2008). Substrate binding mode and its implication on drug design for botulinum neurotoxin A. PLoS Pathog..

[14] Eubanks LM (2007). An *in vitro* and *in vivo* disconnect uncovered through high-throughput identification of botulinum neurotoxin A antagonists. PNAS.

[15] Silvaggi NR (2007). Structures of Clostridium botulinum neurotoxin serotype A light chain complexed with small molecule inhibitors highlight active-site flexibility. Chem. Biol..

[16] Kadkhodayan S, Knapp MS, Schmidt JJ, Balhorn LR (2000). Cloning, expression, and one-step purification of the minimal essential domain of the light chain of botulinum neurotoxin type A. Prot. Expr. Purif..

[17] Ahmed SA (2001). Enzymatic autocatalysis of botulinum A neurotoxin light chain. J. Protein Chem..

[18] Baldwin MR, Bradshaw M, Johnson EA, Barbieri JT (2004). The C-terminus of botulinum neurotoxin type A light chain contributes to solubility, catalysis, and stability. Protein Expr. Purif..

[19] Cai S, Singh BR (2001). Role of the Disulfide Cleavage Induced Molten Globule State of Type A Botulinum Neurotoxin in Its Endopeptidase Activity. Biochemistry.

[20] Kumar R (2014). Botulinum neurotoxin: unique folding of enzyme domain of the most-poisonous poison. J. Biomolec. Struc. Dynam..

[21] Singh BR, DasGupta BR (1989). Molecular differences between type A botulinum neurotoxin and its toxoid. Toxicon.

[22] Ahmed SA, Olson MA, Ludvico ML, Gilsdorf J, Smith LA (2008). Identification of residues surrounding the active site of type A botulinum neurotoxin important for substrate recognition and catalytic activity. Protein J..

[23] Slavik J, Horak J, Rihova L, Kotyk A (1982). Anilinonaphthalene sulfonate fluorescence and amino acid transport in yeast. J. Membr. Biol..

[24] Semisotnov GV (1991). Study of the “molten globule” intermediate state in protein folding by a hydrophobic fluorescent probe. Biopolymers.

[25] Fasshauer D, Bruns D, Shen B, Jahn R, Brunger AT (1997). A structural change occurs upon binding of syntaxin to SNAP-25. J. Biol. Chem..

[26] Bentivoglio AR, Del Grande A, Petracca M, Ialongo T, Ricciardi L (2015). Clinical differences between botulinum neurotoxin type A and B. Toxicon.

[27] Weisemann J (2015). Generation and characterization of six recombinant botulinum neurotoxins as reference material to serve in an international proficiency test. Toxins.

[28] Kukreja R, Singh BR (2015). The botulinum toxin as a therapeutic agent: molecular and pharmacological insights. Res. Rep. Biochem..

[29] Dressler D, Benecke R (2007). Pharmacology of therapeutic botulinum toxin preparations. Disabil. Rehabil..

[30] Krieglstein KG, DasGupta BR, Henschen AH (1994). Covalent structure of botulinum neurotoxin type A: Location of sulfhydryl groups, and disulfide bridges and identification of C-termini of light and heavy chains. J. Prot. Chem..

[31] DasGupta BR, Dekleva ML (1990). Botulinum neurotoxin type A: sequence of amino acids at the N-terminus and around the nicking site. Biochimie.

[32] Lacy DB, Tepp W, Cohen AC, DasGupta BR, Stevens RC (1998). Crystal structure of botulinum neurotoxin type A and implications for toxicity. Nature Struc. Biol..

[33] Li L, Singh BR (2000). Role of zinc binding in type A botulinum neurotoxin light chain’s toxic structure. Biochemistry.

[34] Li L, Singh BR (2000). Spectroscopic analysis of pH-induced changes in the molecular features of type A botulinum neurotoxin light chain. Biochemistry.

[35] Ahmed SA, McPhie P, Smith LA (2003). Autocatalytically fragmented light chain of botulinum A neurotoxin is enzymatically active. Biochemistry.

[36] Cai S, Kukreja R, Shoesmith S, Chang T-W, Singh BR (2006). Botulinum neurotoxin light chain refolds at endosomal pH for its translocation. Protein J..

[37] Feltrup TM, Singh BR (2012). Development of a fluorescence internal quenching correction factor to correct botulinum neurotoxin type A endopeptidase kinetics using SNAPtide. Anal. Chem..

[38] Mizanur RM, Stafford RG, Ahmed SA (2014). Cleavage of SNAP25 and its shorter versions by the protease domain of serotype A botulinum neurotoxin. PloS ONE.

[39] Kumar R, Cai S, Ojadi E, Singh BR (2015). Resolution of sub-nanosecond motions in botulinum neurotoxin endopeptidase: An evidence of internal flexibility. Biochim. Biophys. Acta.

[40] Silhar P (2012). C-terminus of botulinum A protease has profound and unanticipated kinetic consequences upon the catalytic cleft. ACS Med. Chem. Lett..

[41] Eichorn T, Dolimbek BZ, Deeg K, Efferth T, Atassi MZ (2012). Inhibition *in vivo* of the activity of botulinum neurotoxin A by small molecules selected by virtual screening. Toxicon.

[42] Teng YH (2015). Computer-aided identification, synthesis, and biological evaluation of novel inhibitors for botulinum neurotoxin serotype A. Bioorg. Med. Chem..

[43] Singh BR, DasGupta BR (1989). Structure of heavy and light chain subunits of type A botulinum neurotoxin analyzed by circular dichroism and fluorescence measurements. Mol. Cell. Biochem..

[44] Eubanks LM (2010). Identification of a natural product antagonist against the botulinum neurotoxin light chain protease. ACS Med. Chem. Lett..

[45] Silhar P (2013). Evaluation of adamantine hydroxamates as botulinum neurotoxin inhibitors: synthesis, crystallography, modeling, kinetic and cellular based studies. Bioorg. Med. Chem..

[46] Seki H (2015). Toward the discovery of dual inhibitors for botulinum neurotoxin A: concomitant targeting of endocytosis and light chain protease activity. Chem. Commun..

[47] Shoemaker BA, Portman JJ, Wolynes PG (2000). Speeding molecular recognition by using the folding funnel: The fly-casting mechanism. PNAS.

[48] Breidenbach MA, Brunger AT (2004). Substrate recognition strategy for botulinum neurotoxin serotype A. Nature.

[49] Tompa P (2011). Unstructured biology coming of age. Curr. Opin. Struc. Biol..

[50] Uversky VN (2013). Unusual biophysics of intrinsically disordered proteins. Biochim. Biophys. Acta.

[51] Tompa P (2005). The interplay between structure and function in intrinsically unstructured proteins. FEBS Lett.

[52] Uversky VN (2010). Multitude of binding modes attainable by intrinsically disordered proteins: a portrait gallery of disorder-based complexes. Chem. Soc. Rev..

[53] Sickmeier M (2007). DisProt: the database of disordered proteins. Nuc. Acids Res..

[54] Dunker AK, Brown CJ, Lawson JD, Iakoucheva LM, Obradovic Z (2002). Intrinsic Disorder and Protein Function. Biochemistry.

[55] Babu MM, Lee RV, Groot NS, Gsponer J (2011). Intrinsically disordered proteins: Regulation and disease. Curr. Opin. Struct. Biol..

[56] Feltrup, T. M., Kumar, R. & Singh, B. R. Relevance of intrinsic disorder in protein structure and function, in *Protein Toxins in Modeling* Biochemistry (eds Kumar, R. & Singh B. R.) 1^st^ ed., pp 29–72, Springer Briefs in Biochemistry and Molecular Biology, ISBN 978-3-319-43538-1 (2016).

[57] Li L, Singh BR (1999). Structure-Function Relationship of Clostridial Neurotoxins. J. Toxicol – Toxin Rev..

[58] Whitaker JR, Granum PE (1980). An absolute method for protein determination based on difference in absorbance at 235 and 280 nm. Anal. Biochem..

[59] Kuo C-K, Oyler G, Shoemaker CB (2009). Lipid and cationic polymer based transduction of botulinum holotoxin or toxin protease alone, extends the target cell range and improves the efficiency of intoxication. Toxicon.

[60] Janardhanan P, Ravichandran E, Cai S (2015). RNA aptamer as potential antidote against botulism: An *in vivo* report. Botulinum J..

