# Supplementary material for: A novel role of C-terminus in introducing a functionally flexible structure critical for the biological activity of botulinum neurotoxin
Source: Sci Rep. 2018 Jun 11;8:8884. doi: 10.1038/s41598-018-26764-z (PMC5995822; doi:10.1038/s41598-018-26764-z)
Supplement: Supplementary file 1 — Supplementary Information [file 41598_2018_26764_MOESM1_ESM.docx]

**SUPPLEMENTARY INFORMATION**

**A novel role of C-terminus in introducing a functionally flexible structure critical for the**

**biological activity of botulinum neurotoxin**

*Thomas M. Feltrup^†^, Kruti Patel^†^, Raj Kumar^‡^, Shuowei Cai^†^, and Bal Ram Singh^‡*^*

^†^ Department of Chemistry & Biochemistry, University of Massachusetts Dartmouth, North Dartmouth, MA 02747

^‡^­Botulinum Research Center, Institute of Advanced Sciences, North Dartmouth, MA 02747

**fLCA**

**tLCA**

**Control**

**fLCA**

**tLCA**

**Control**

**fLCA**

**tLCA**

**Control**

**Figure S1.** Full Western blot image of SNAP-25 cleavage in M17 neuroblastoma cells by LCA. Unprocessed blot of triplicate samples run on the same Western blot is shown with lanes labeled as: Control (M17 cells received no LCA treatment); tLCA (M17 cells treated with 50 nM tLCA); fLCA (M17 cells treated with 50 nM fLCA). Data corresponds to Western blot shown in Fig. 1b.

**a**

**b**


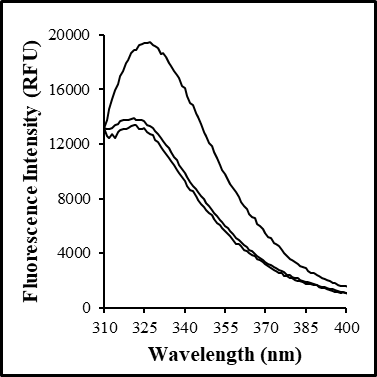

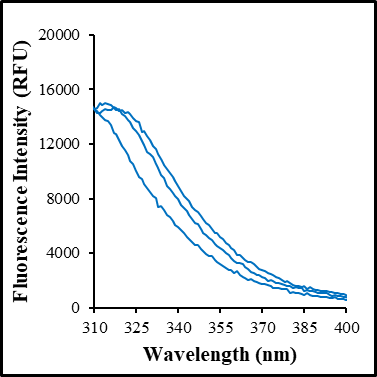


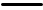

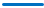


**Figure S2.** Comparison of three independent emission spectra of **a.** fLCA ( ) and **b.** tLCA ( ) using a 295 nm λ_ex_ at 25 ^o^C. The average λ_max_ was calculated using the λ_max_ of each of the three spectra.

**Figure S3.** LCA-bound ANS emission spectra at 25 ^o^C and 37 ^o^C. **A.** Shown are ANS bound with fLCA (
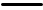
) and tLCA (
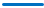
) at 25 ^o^C. **B.** Shown are ANS bound with fLCA (
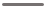
) and tLCA (
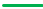
) at 37 ^o^C. Each spectra is an average of at least three samples.


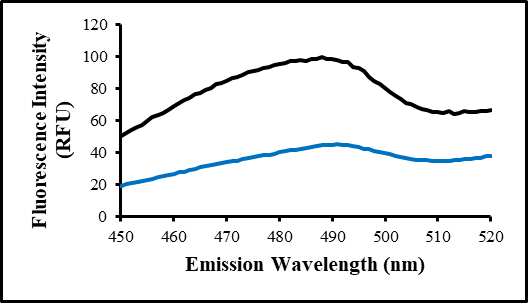

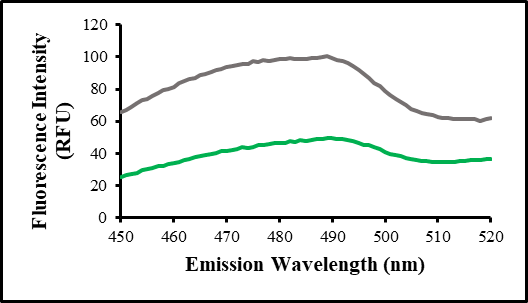


**a**

**b**

**Figure S4.** Far-UV CD spectra of sn2 (dissolved in 10 mM sodium phosphate, pH 7.5 containing 150 mM NaCl) at 25 ^o^C. Spectrum is an average of three samples.


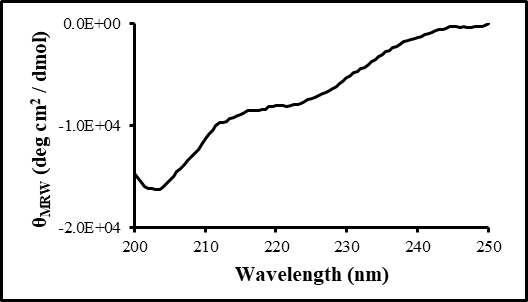

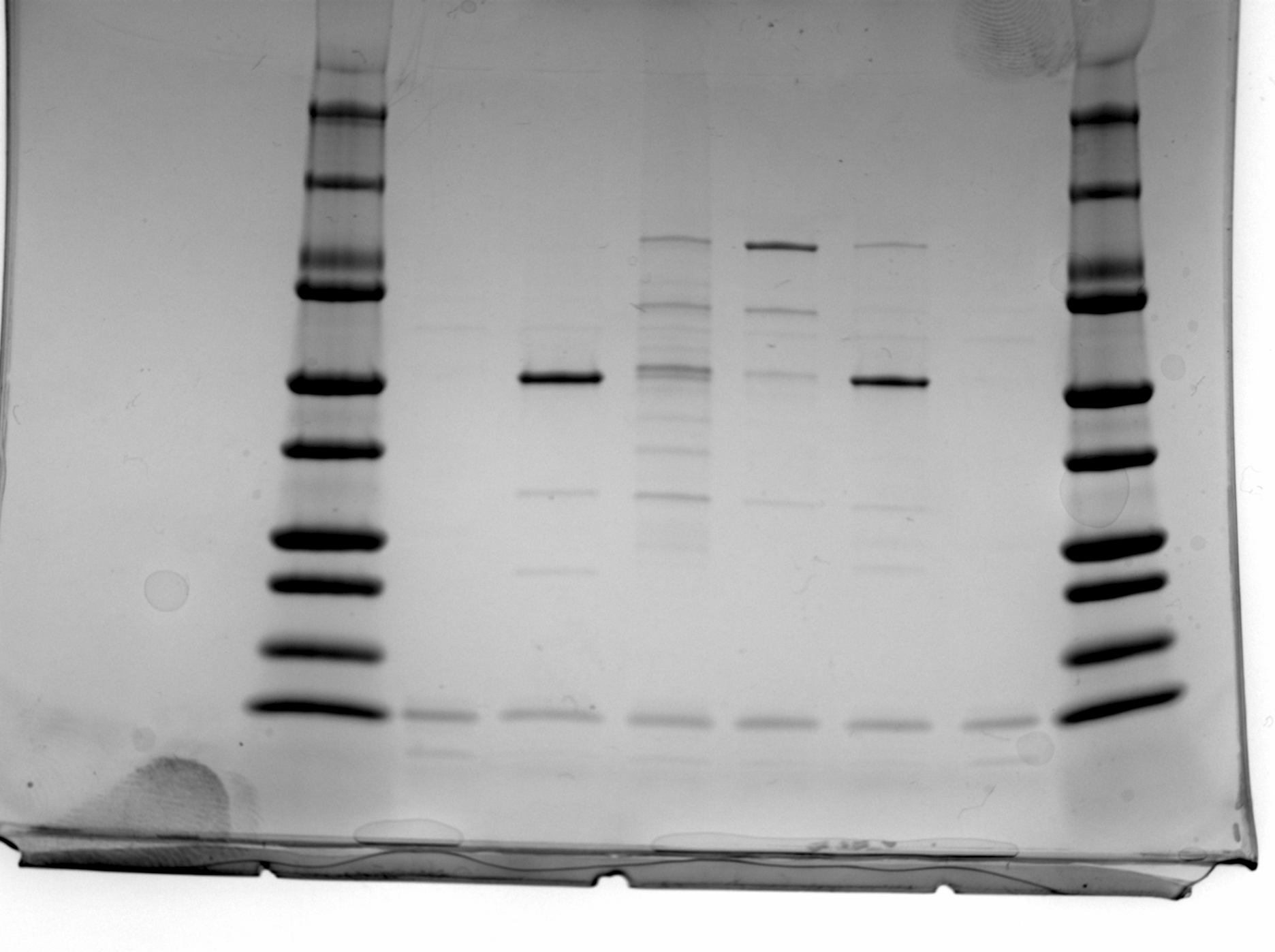


10 kDa

Lane 1

Lane 2

Lane 3

Lane 4

**Figure S5.** Incubation of cleavage-resistant SN2 (0.2 mg/mL) and wtLCA or tLCA (0.2 mg/mL). Samples were incubated at 25 ^o^C for 15 min followed by a 15 min incubation at 37 ^o^C to simulate the conditions each LCA would be exposed to during CD measurements. The SN2 band remained in-tact throughout this series of incubations.

Lane identification – Lane 1: Broad range marker; Lane 2: control SN2 with no LCA; Lane 3: SN2 incubated with tLCA; Lane 4: SN2 incubated with fLCA.

**Figure S6.** SPR sensorgrams obtained from CM3 chip with immobilized SN2 on the surface. SPR sensorgram obtained when **a.** fLCA and **b.** tLCA at 2.5, 5, 10, 20, 40, 60, 89, 133, 200, and 300 nM (from bottom to top) were injected onto an SN2-immobilized CM3 sensor chip with a flow rate of 10 µl/min. The response unit (in RUs) were recorded as a function of time.

**b**

**a**
